# Dysregulation of Histone Acetyltransferases and Deacetylases in Cardiovascular Diseases

**DOI:** 10.1155/2014/641979

**Published:** 2014-02-18

**Authors:** Yonggang Wang, Xiao Miao, Yucheng Liu, Fengsheng Li, Quan Liu, Jian Sun, Lu Cai

**Affiliations:** ^1^Cardiovascular Center, The First Hospital of Jilin University, 71 Xinmin Street, Changchun 130021, China; ^2^Department of Pediatrics, Kosair Children Hospital Research Institute, University of Louisville, 570 South Preston Street, Baxter I, Suite 304F, Louisville, KY 40202, USA; ^3^The Second Hospital of Jilin University, Changchun 130041, China; ^4^The Second Artillery General Hospital, Beijing 100088, China

## Abstract

Cardiovascular disease (CVD) remains a leading cause of mortality worldwide despite advances in its prevention and management. A comprehensive understanding of factors which contribute to CVD is required in order to develop more effective treatment options. Dysregulation of epigenetic posttranscriptional modifications of histones in chromatin is thought to be associated with the pathology of many disease models, including CVD. Histone acetyltransferases (HATs) and deacetylases (HDACs) are regulators of histone lysine acetylation. Recent studies have implicated a fundamental role of reversible protein acetylation in the regulation of CVDs such as hypertension, pulmonary hypertension, diabetic cardiomyopathy, coronary artery disease, arrhythmia, and heart failure. This reversible acetylation is governed by enzymes that HATs add or HDACs remove acetyl groups respectively. New evidence has revealed that histone acetylation regulators blunt cardiovascular and related disease states in certain cellular processes including myocyte hypertrophy, apoptosis, fibrosis, oxidative stress, and inflammation. The accumulating evidence of the detrimental role of histone acetylation in cardiac disease combined with the cardioprotective role of histone acetylation regulators suggests that the use of histone acetylation regulators may serve as a novel approach to treating the millions of patients afflicted by cardiac diseases worldwide.

## 1. Introduction

Heart failure remains a leading cause of the mortality world-wide, which is attributed to cardiovascular diseases, such as coronary artery disease, hypertension, and diabetic cardiomyopathy. Cardiac remodeling, defined as alterations in the structure of the heart in response to hemodynamic load and/or cardiac injury in association with neurohormonal activation, is generally accepted as a determinant of the clinical course of heart failure [1]. The causes of cardiac remodeling share common molecular, biochemical, and mechanical pathways and are associated with a number of cellular changes including myocyte hypertrophy, contractility [2, 3], loss of myocytes through apoptosis [4], fibrosis [5–7], oxidative stress [8], autophagy [9], inflammation [10], and energy metabolism [11].

A consensus definition of an epigenetic trait, ‘‘stably heritable phenotype resulting from changes in a chromosome without alterations in the DNA sequence,” was reached at a Cold Spring Harbor meeting [12]. Simply put, epigenetic changes are able to modulate the activation of certain genes, without affecting the sequence of DNA. Eukaryotic DNA is highly organized and packaged into the nucleus. The organization and packaging of DNA are achieved through the addition of proteins-histones. Histones have long N-terminal tails and core histones H2A, H2B, H3, and H4; together with DNA, histones form a complex structure called chromatin. The modification of core histones is very important to conformational changes of the chromatin. Core histones are subject to diverse posttranslational modifications such as acetylation, methylation, phosphorylation, ubiquitination, and sumoylation. One of the best characterized posttranslational modifications is acetylation, which occurs at the *ε* amino groups of lysine residues in the core histone. Acetylation of core histones plays a central role in the epigenetic regulation of gene expression in eukaryotic cells, which is tightly regulated by two opposing families of proteins, HATs and HDACs. Recent studies have demonstrated that acetylation of core histone is associated with many cardiovascular diseases (CVDs), including hypertension [13, 14], diabetic cardiomyopathy [15], myocardial infarction [16], pulmonary arterial hypertension [17, 18], and cellular disorders, such as vascular smooth muscle cell proliferation [19] and apoptosis [20].

In this review, we will summarize (1) enzymatic regulation of acetylation level in the heart, (2) the functional effect of acetylation on the cellular processes involved in cardiac remodeling, and HAT and HDAC status in the heart, (3) current pharmaceutical approaches available to regulate acetylation and (4) finally, future directions that will be discussed.

## 2. Enzymes Regulating Acetylation Levels

Protein lysine acetylation was first reported nearly 50 years ago [21]. Nucleus-localized transcription factors and its co-regulators are most well characterized acetylation substrates [22, 23]. Transcription activators and repressors can recruit HATs or HDACs, respectively, to regulate transcription. Studies have demonstrated a critical role of acetylation in regulating chromatin structure and gene expression [24, 25]. Acetylation and deacetylation of core histones in chromatin are the most important types of histone modifications and are essential for many biological processes [26]. In general, HATs induce transcriptional activation by adding acetyl groups while HDACs induce transcription inhibition by removing acetyl groups from conserved lysine residues within the N-terminal tails of histones H3 and H4 [27].

### 2.1. HATs

**Figure 1:**
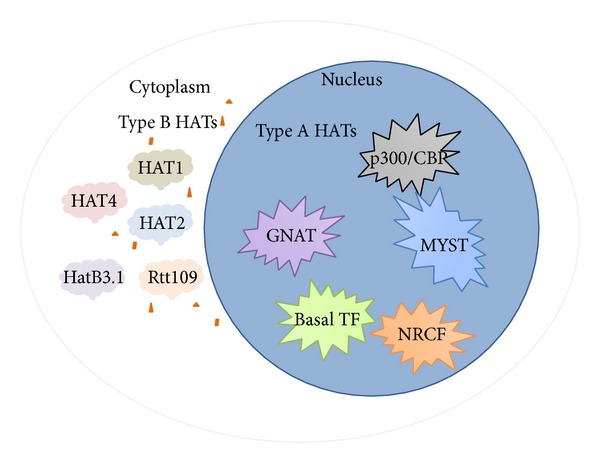
HATs classifications. HATs are categorized into two types: type A and type B; type A are nuclear HATs; type B are cytoplasmic HATs. Type A HATs are further divided into five families: GNAT family, p300/CBP family, MYST family, basal TF family, and NRCF family. Type B HATs are further divided into HAT1, HAT2, HatB3.1, Rtt109, and HAT4.

Briefly, HATs are divided into two types, nuclear (type A HATs) and cytoplasmic (type B HATs). Type A HATs are transcription related and subclassified into five families: (1) GNAT (GCN5-related acetyltransferases) family represented by GCN5 (General Control Nonderepressible), PCAF (p300/CBP associated factor), and ELP3 (Elongator complex protein 3); (2) p300/CBP family represented by p300 and CBP; (3) MYST (MOZ, YBF2/SAS3, SAS2, and TIP60 protein) family, which consists of MYST1 (HMOF, males absent on the first), MYST2 (HBO1, histone acetyltransferase binding to ORC), MYST3 (MOZ, monocytic leukemia zinc finger), MYST4 (MORF, monocytic leukemia zinc finger protein-related factor), and TIP60 (tat interacting protein 60 kDa); (4) basal TF family; TFIIIC (Transcription Factor IIIC), TAF1, and (5) finally, NRCF (nuclear receptor cofactors) family, SRC (steroid receptor coactivator) ACTR/NCOA3 (nuclear receptor coactivator 3). The cytoplasmic type B HATs are responsible for deposition-related acetylation of free histone substrates only a few B-HATs have been characterized, including HAT1, HAT2 [28], Rtt109 [29], HatB3.1 [30], and HAT4 [31]. For clarity, each of these systems will be addressed separately in Figure 1. The most extensively studied HATs in muscle are p300 and the closely related coactivator, CREB-binding protein (CBP), which play critical roles in physiological and pathological growth of cardiac myocytes. The p300 possesses intrinsic HAT activity that modifies chromatin and associated transcription factors, thereby relaxing chromatin structure and promoting gene activation. The role of p300 in normal cardiac transcription is demonstrated by the phenotype of p300 knockout mice, which die between days 9 and 11.5 of gestation. In addition, those mice also show reduced expression of muscle structural proteins such as *β*-myosin heavy chain (MHC) and *α*-actinin, as well as cardiac structural defects and reduced trabeculation [32]. Further, a gene knock-in study demonstrated the importance of the HAT domain of p300 in heart formation [33].

### 2.2. HDACs

**Figure 2:**
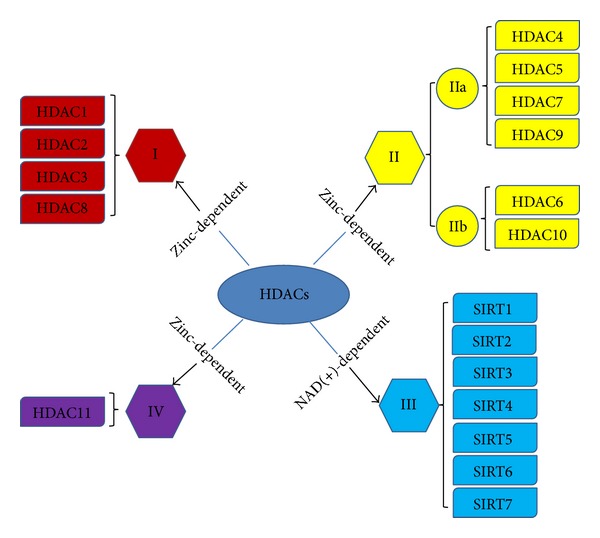
HDACs classifications. HDACs have two subclasses: zinc-dependent and NAD(+)-dependent, which are further divided into four major classes: class I HDACs (HDAC1, 2, 3, and 8); class II HDACs are divided into IIa (HDAC4, 5, 7, and 9) and IIb (HDAC6 and 10); class III HDACs (SIRT1-7); and class IV (HDAC11).

There are four major classes of HDACs that have been identified in mammals. class I HDACs (HDAC1, 2, 3, and 8) are widely expressed and consist mainly of a catalytic domain surrounded by short NH2 and COOH termini; class II HDACs are further divided into two subclasses, which are IIa (HDAC4, 5, 7, and 9) and IIb (HDAC6 and 10), which contain one or two catalytic sites [22]. Class III HDACs are sirtuins (SIRT1-7) and class IV consists of a solitary member HDAC11, which is homologous to Rpd3 and Hda1 proteins of yeast (Figure 2) [34]. It has been shown that many HDACs have a highly conserved domain; recent studies, however, showed that class I and IIa HDACs have opposing roles in regulating cardiac hypertrophy. Evidence for the mechanisms by which the distinct classes of HDACs act to control cardiac hypertrophy is accumulating [35].

## 3. Effects of Abnormal Acetylation in Cardiovascular Disease

A global acetylation level change is observed in many human diseases. The relationship between acetylation and cancer, such as renal cell carcinoma [36] and breast cancer [37], is becoming more apparent. Similar findings have been reported in nervous system diseases [38, 39]. Most cancers and nervous system diseases are caused by environmental factors as well as genetic predisposition. In addition, recent studies suggest that CVD and diabetes are significantly associated with exposure to environmental chemicals present in air, food, and water. These relationships are likely the consequence of the combination of epigenetic effects (including acetylation and deacetylation) and gene induction [40]. Recently, some studies showed that acetylation level is associated with cardiovascular disease, such as hypertension [13, 14], diabetic cardiomyopathy [15], coronary artery disease (CAD) [16], arrhythmia [41], pulmonary arterial hypertension (PAH) [17, 18], and heart failure [42].

### 3.1. Hypertension and Pulmonary Arterial Hypertension (PAH)

Hypertension is a systemic vascular disease, an epidemic health concern, and a major risk factor for the development of CVD. It has long been established that high blood pressure is often accompanied by increased activation of the renin-angiotensin system (RAS) and the sympathetic nervous system (SNS). Environmental changes could have an effect on blood pressure that leads to the epigenetic modifications. PAH is a progressive disease characterized by pulmonary vascular remodeling and right heart failure. The disease remains a life-limiting condition with a major impact on the patient's quality of life. Over several decades, increasing evidence suggested that epigenetic mechanisms, especially aberrant acetylation of histone, contributed to the development of hypertension and PAH, which implied that pharmacological therapies targeting the regulator of acetylation might be a novel approach for the treatment of hypertension and PAH. It has been approved that the inhibition of HDAC can attenuate hypertensive responses in spontaneously hypertensive rats [43]. Using the HDAC inhibitor, valproic acid (VPA), Dr. Lee further found that HDAC inhibition prevented the development of hypertension through attenuating transcriptional activity of mineralocorticoid receptor (MR) by increasing its acetylation [13]. MR acetylation reduced hormone-responsive element binding affinity, RNA polymerase recruitment, and expression of target genes, which in turn regulate intracellular salt balance [13]. Furthermore, Lacolley et al. observed that smooth muscle cells (SMCs) play a significant role during hypertension-associated structural vascular changes [44]. It is well known that angiotensin II (Ang II) is the bioactive peptide in the RAS and plays a critical role in hypertension. It was found that SMCs treated with Ang II showed elevated phosphorylation of HDAC5 at serine 259/498 in a time and dose-dependent manner. Serine phosphorylation stimulated nuclear export of HDAC5, which was associated with reduced Ang II-induced myocyte enhancer factor-2 (MEF2) transcriptional activity and protein synthesis [45]. In addition to HDAC5, HDAC4 is another factor which may be involved in Ang II-induced SMC hypertrophy. Ang II treatment results in phosphorylation through calmodulin kinase II and nuclear export of HDAC4, resulting in augmentation of the hypertrophic transcription factor MEF2 [46]. Finally, treatment of spontaneously hypertensive rats with the HDAC inhibitor, trichostatin A (TSA), effectively reduced blood pressure and vascular inflammation [47]. In PAH, epigenetic mechanism has been considered to mediate environment-induced altering gene expression [48]. Xu et al. observed that hypoxia was strongly correlated with increased histone acetylation and hypoxia inducible factor-1*α* (HIF-1*α*) binding levels in the endothelin-1 (ET-1) gene core promoter region in a hypoxic pulmonary hypertension rat model, further indicating the significance of epigenetic modifications in PAH and pulmonary vascular remodeling [49]. One article describes how HDAC1 and HDAC5 protein levels were elevated in PAH and right ventricles from rats exposed to hypoxia. Two HDAC inhibitors [VPA and suberoylanilide hydroxamic acid (SAHA)] were used to treat the rats, which resulted in augmented histone acetylation levels by both SAHA and VPA treatments. HDAC inhibitors prevent constitutive growth of adventitial fibroblasts (PH-Fibs) and R-Cells as well as platelet-derived growth factor (PDGF)-induced human pulmonary SMCs proliferation [50]. Li et al. demonstrated that alterations in HDACs contribute to a proinflammatory phenotype of PH-Fibs (increased migration, adhesion and activation of monocytes). Further, they determined that inhibition of HDACs results in attenuation of PH-Fibs functional activity [51]. Isoform-selective HDAC inhibitors have been shown to have beneficial effects on the right ventricle via inhibition of pathological gene expression, inhibition of proapoptotic caspase activity, and repression of pro-inflammatory protein expression in hypobaric hypoxia rats [52].

The results of these studies warrant further investigation as histone hyperacetylation is correlated with reduced expression of factors involved in vascular injury and remodeling. A novel approach to evaluate receptor function such as MR [13], or epigenetic modulations [53], would enable early diagnosis of organ damage and individualized therapy.

### 3.2. Atherosclerosis and Coronary Artery Disease (CAD)

Atherosclerosis is initiated by endothelial dysfunction and lipid accumulation in the vessel wall, resulting in the formation of fatty streak lesions [54]. Sometimes called hardening of the arteries, atherosclerosis can slowly narrow and harden the arteries throughout the body. Atherosclerosis is the primary cause of CAD, which occurs when atherosclerosis affects the arteries of the heart. CAD is the major cause of death worldwide [55]. Most of these deaths occur as a result of heart attacks, caused by blood clots in the arteries of the heart.

It has been reported that there is a connection between acetylation status and atherosclerosis. Modulation of HAT activity derived from CBP through extracellular thrombin-signaling via the mitogen-activated protein kinases (MAPKs) pathway in vascular smooth muscle cells (VSMCs), suggests that this pathway causes hypernuclear acetylation in atherosclerotic lesions [56]. Recently, many studies were published, directly linking epigenetic histone modifications and atherosclerosis. SMCs constitute the most abundant cell type in the arterial wall and are involved in all stages of atherosclerosic lesion formation. SMCs have been demonstrated to contribute to atherosclerotic plaque formation through migration, proliferation, matrix synthesis, apoptosis, inflammation, and foam cell formation through cholesterol uptake [57]. *In vitro* studies of SMC differentiation have revealed the ability of serum response factor (SRF) and the SRF-cofactor, myocardin, to bind to and activate CArG-elements of SMC marker genes is preceded and dependent on specific histone hyperacetylation, which is likely mediated by HATs [58–60]. It has been shown that neointimal lesions in atherosclerosis-prone Ldlr (−/−) mice were exacerbated by the treatment of the pharmacologic HDAC inhibitor, TSA [61]. These results emphasize the need for a clear understanding of histone core pathways in animal models of atherosclerosis. The association between DNA methylation and CAD is well documented in recent twin studies [62, 63]. In this review, we focus on the relationship of acetylation and CAD. HDAC activity may also play a significant role in determining the severity of myocardial ischemia and reperfusion damage, particularly following myocardial infarction (MI). Inhibition of HDACs by TSA treatment in cultured embryonic stem cells stimulates myogenesis and angiogenesis, indicating that HDAC inhibition can stimulate angiogenesis and thus minimize a loss in myocardial performance after MI [35]. These studies raise the possibility that HDAC inhibition could be used, initially, as an infarct-size reducing strategy and subsequently as a long-term treatment option to inhibit postinfarct remodeling by mobilizing cardiac stem cells. Acute ischemic preconditioning (IPC) induces protection against cardiac ischemia-reperfusion (IR) via post-translational modification of key proteins. It was found that Lys deacetylation occurs during IPC and an elevation in SIRT1 activity plays a role in its protective effect in the heart [64]. SIRT1 activity is increased in IPC, but SIRT1 protein levels do not change; inhibition of either SIRT1 or Nampt inhibits IPC-mediated deacetylation and hence blunts the cardioprotection conferred by IPC. During IPC, deacetylation of cardiomyocyte cytosolic proteins is likely mediated by SIRT1 [64].

Precise determination of histone modifications at specific genes may prove to be valuable in understanding gene expression profiles in vascular disease, particularly since small molecules can inhibit the function of histone modifying enzymes, altering the expression of genes [65]. Therefore, intervention in epigenetic gene regulation has potential to be an effective therapeutic intervention, especially in complex multifactorial diseases such as atherosclerosis and CAD.

### 3.3. Cardiomyopathy and Heart Failure

Heart failure is a common, costly, disabling, and potentially deadly condition. Common causes of heart failure include ischemic heart disease, hypertension, valvular heart disease, and cardiomyopathy [66]. Many signaling pathways lead to remodeling observed in heart failure, which is the end-stage of all heart diseases. Pathological ventricular remodeling leading to heart failure is the combination of a complex series of transcriptional, signaling, structural, electrophysiological, and functional events occurring within the cardiac myocyte. Kehat and colleagues briefly discussed remodeling as a complex phenomenon composed of both adaptive and maladaptive responses of cardiomyocytes and surrounding cells [67]. Acetylation is proved to be involved in the cardiac remodeling process. During the last decade, the role of several HATs and HDACs in heart disease has been studied. Gene deletion and overexpression studies have found that these enzymes play significant roles in the pathological processes of cardiac remodeling, including hypertrophy, apoptosis, necrosis, metabolism, contractility, and fibrosis [42].

During agonist-induced hypertrophy of cardiomyocytes, p300 transcriptional activity is enhanced [68]. In addition, ectopic overexpression of p300 and CBP stimulate cardiac growth, while dominant-negative mutants of p300 block agonist-mediated cardiac growth [69, 70]. Transgene-mediated expression of p300 in the adult mouse heart also causes hypertrophy and heart failure [70]. In addition to acetylation of histone tails, p300 also serves as an adaptor for hypertrophy-responsive transcription factors, such as GATA4, SRF, and MEF2; these interactions are required for full transcriptional activity of these factors [71–73]. The activity of p300/CBP is enhanced by signaling pathways that promote cardiac hypertrophy, mediating the stimulation of the fetal gene program by hypertrophy-inducing factors.

There are many pathological cellular processes which promote heart remodeling, such as inflammation, apoptosis, cell proliferation, oxidative stress, and fibrosis. Acetylation also plays a crucial role with respect to remodeling. The impact of HDAC inhibition on inflammation in heart failure models was recently addressed. In spontaneously hypertensive rats, animals treated with the HDAC inhibitor, VPA, for 20 weeks showed reduced expression of IL-1*β* and TNF*α* in left ventricle, which is associated with reduced cardiac hypertrophy and fibrosis and resulted in improved cardiac function [74]. Dingar and colleagues utilized chromatin immunoprecipitation to determine that the E2F transcription factor 4 (E2F4)-p130-repressor directly blocks transcription of essential apoptosis-related genes, E2F1, Apaf-1, and p73*α*, through recruitment of HDAC1 causing apoptosis. Expression of HDAC1, H141A, or HDAC-binding deficient p130ΔHDAC1 abolishes the anti-apoptotic effect of E2F4* in vitro *and* in vivo *[75]. The utility of selective small-molecule inhibitors of class I HDACs was assessed in a preclinical model of pulmonary hypertension, where it was determined that selective class I histone deacetylase inhibition suppresses hypoxia-induced cardiopulmonary remodeling through an antiproliferative mechanism [52]. Tomita et al. have demonstrated that HDAC inhibitors such as TSA induced hyperacetylation of histone H4 and stimulated expression of the pro-inflammatory cytokine IL-8. These new findings suggest that detection of acetylated histone residues at the single cell level may be a powerful tool to analyze the modulation of cell proliferation and gene transcription [76]. Ventricular fibrosis caused by accumulation of collagen and other extracellular matrix components is a cardinal feature of left ventricular hypertrophy and post-infarct remodeling. Other than aldosterone antagonists, there are currently nontherapeutic agents that effectively reduce cardiac fibrosis [77] in clinical setting.

Planavila et al. observed that Sirt1 deficiency leads to dilated cardiomyopathy in adult mice. Sirt1 null mice exhibit an altered pattern of MEF2 acetylation, and further studies found that Sirt1 regulates MEF2 acetylation status through p300 in the heart [78]. Hypertrophic and apoptotic signaling pathways are increased in the myocardium of Sirt7-null mice as well as in primary Sirt7-deficient cardiomyocytes. Sirt7 increases stress resistance of cardiomyocytes and prevents apoptosis as well as inflammatory cardiomyopathy in mice [79]. Kim et al. demonstrated that HDAC activity is an important factor that affects vertebrate heart tube formation by activating the Wnt/*β*-catenin signaling pathway, which induces bmp4 expression in atrioventricular canal (AVC) myocardial cells [26].

Diabetic cardiomyopathy refers to the changes in contractility that occur to the diabetic heart that can arise in the absence of vascular disease. Clinical trials have shown that histone acetylation is significantly elevated in complication-free diabetic subjects only, suggesting that histone acetylation in this population may be a protective mechanism in the heart [80]. This observation was further confirmed using *in vivo* and *in vitro* models. Hyperglycemic memory may explain why intensive glucose control has failed to improve cardiovascular outcomes in patients with diabetes. p66 (Shc) is the key effector driving vascular hyperglycemic memory in diabetes. Paneni et al. determined that repression of p66 (Shc) expression by SIRT1 contributes to the protection of hyperglycemia-induced endothelial dysfunction [81]. Yu et al. observed that high levels of glucose induced apoptosis in cardiomyocytes via epigenetic regulation of the insulin-like growth factor receptor (IGF-1R). They determined that high glucose can increase the association of p53 with HDAC1 and decrease the association of acetylated H4 with the IGF-1R promoter. Furthermore, the HDAC inhibitor TSA blocked the inhibition of IGF-1R in cardiomyocytes exposed to high glucose conditions [82].

Both class I and class II HDACs expressions have been shown to be associated with cardiac hypertrophy [83]. The class I HDACs, HDAC1 and HDAC2 are expressed ubiquitously, and systemic deletion of either results in embryonic lethality. Class I HDACs are generally considered detrimental to cardiac function [42, 84]. Class I HDACs may induce cardiac hypertrophy via the suppression of antihypertrophic pathways. Cardiac overexpression of HDAC2 also results in hypertrophy by modulating the PI3 K-Akt-Gsk3*β* pathway [85]. Interestingly, transgenic overexpression of HDAC3 in the heart does not induce hypertrophy although it increases postnatal cardiac myocyte proliferation [86]. Singh et al. determined that HDAC3 plays a critical and specific regulatory role in the formation of the cardiac outflow tract [87]. HDAC6 and HDAC8 enzyme activities contribute to cardiac hypertrophy and fibrosis in the heart obtained from chronic hypertensive rats [88].

On the other hand, class II HDACs prevent cardiac hypertrophy by blocking the activity of several prohypertrophic transcription factors, including SRF, GATA4, nuclear factor of activated T-cells (NFAT), and myocardin [89]. The initial interest in studying HDACs in the heart resulted from the discovery that class IIa HDACs interact with members of the MEF2 transcription factor family [90], which also plays an important role in the development of cardiac hypertrophy. Overexpression of class IIa HDACs 4 [91], 5 [92–94], or 9 [95] coordinately suppresses MEF2 dependent transcription and agonist-dependent hypertrophy of cultured cardiac myocytes. In contrast, disruption of the gene encoding HDAC9 in mice leads to increased cardiac MEF2 activity [95]. Mouse knockout models for HDAC5 [96] or HDAC9 [95] develop profound cardiac hypertrophy in response to pressure overload and spontaneous, pathologic hypertrophy with advanced age. HDAC7 has been extensively studied in hypoxia and COPD models. HDAC7 knockdown inhibited hypoxia-induced HIF-1*α* activity in U937 cells and HIF-1*α* nuclear translocation as well as HIF-1*α* binding to the VEGF promoter in A549 cells. However, HDAC7 has not been extensively studied in the context of CVD [97, 98]. The results of these studies support a role for class IIa HDACs as endogenous inhibitors of cardiac hypertrophy.

**Figure 3:**
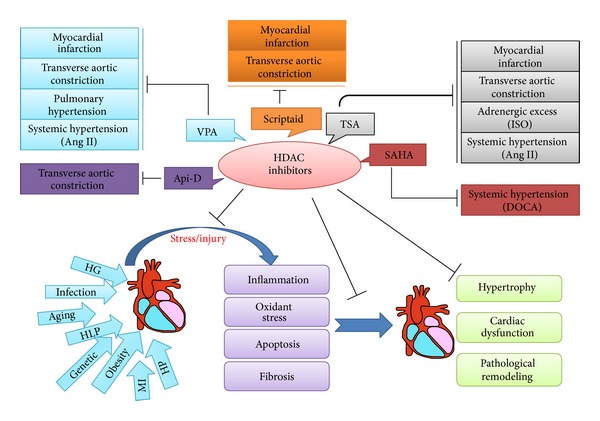
The protective effect of HDAC inhibitors in cardiac remodeling. Many risk factors such as high glucose (HG), myocardial infarction (MI), hypertension (HP), hyperlipidaemia (HLP), genetic causes (cardiomyopathy), obesity, and aging cause cardiac injury, activate pathological cellular processes (inflammation, apoptosis, oxidative stress, and fibrosis), and induce cardiac hypertrophy, remodeling, and dysfunction. HDAC inhibitors are capable of blocking elements of these detrimental biological processes and preserving cardiac function. The HDAC inhibitors, trichostatin A (TSA), suberoylanilide hydroxamic acid (SAHA), valproic acid (VPA), scriptaid, and apicidin-derivative (Api-D) have been tested in rodent heart failure models.

A hypertrophic response is also observed in the heart as a result of increased stress associated with chronic hypertension, injury (myocardial infarction), genetic causes (cardiomyopathy), infection (myocarditis), valvular heart disease, obesity, diabetes, and aging [99]. Most research related to the epigenetics of heart failure has focused on histone acetylation. Taken together, these studies suggest that different classes of HDACs are involved in different pathways that control heart remodeling (Figure 3).

These studies highlight an important role of histone modifications in pathways linked to CVD development, although further studies are warranted, particularly concerning clinical implications of histone modulating enzymes such as HDAC inhibitors for therapeutic strategies in heart disease [100].

### 3.4. Redox/Oxidative Metabolism Controlling HDAC and HAT Function in Cardiovascular Disease

In the above sections, we have discussed the effect of HDAC and HAT function in various CVDs. It is appreciated that oxidative stress plays a critical role in the initiation and progression of these CVD; therefore, what is the relationship of oxidative stress with the abnormal function of HDACs and HATs has been recently interesting for investigators [101, 102]. Reportedly moderate levels of reactive oxygen species (ROS) are required for some signaling pathways, whereas exceedingly high levels of it may also cause certain molecule acetylation indirectly. For instance, the p53 tumor suppressor gene is activated in response to oxidative DNA damage resulting in either growth arrest or apoptosis. The p53 positive regulator, homeodomain interacting protein-kinase 2 (HIPK2) as a nuclear serine/threonine kinase, regulates p53-dependent apoptosis through selective p53 phosphorylation at serine 46 and beyond serine 46 phosphorylation. For the latter, HIPK2 was found to promote p53 acetylation by cooperating with the HAT protein PCAF [103]. It is known that HIPK2 can be modified by small ubiquitin-like modifier (SUMO), called sumoylation. Sumoylation of HIPK2 at permissive ROS levels allows the constitutive association of HDAC3 with HIPK2 and keeps HIPK2 in the nonacetylated state. Sumoylation is a highly dynamic process, and SUMO attachment is readily reversed by sentrin-specific proteases (SENPs). Therefore, whether SUMO can modify HIPK2 is affected by ROS levels since high levels of it can block sumoylation probably due to the reversible formation of disulfide bonds between catalytic cysteines of the SUMO E1 subunit Uba2 and the E2-conjugating enzyme Ubc9 [104] or by ROS-mediated stabilization of SENP [105]. Elevated ROS levels prevent sumoylation of HIPK2 and, consequently, reduce association of HDAC3, thus leading to the acetylation of HIPK2. Therefore, HIPK2 acetylation was considered as a molecular switch to leave the basal activities of HIPK2 intact but restrict its ability to trigger ROS-mediated cell death. ROS-inducible HIPK2 acetylation is regulated by a molecular mechanism that employs redox-dependent desumoylation of HIPK2, which in turn impairs HIPK2/HDAC3 binding and thus allows for HIPK2 acetylation [106]. The above studies indicate that ROS are involved in HIPK2 and P53 activity regulation via multiple mechanisms, including chromatin remodeling.

In addition, reportedly there was also a new post-translational regulation of mitochondria manganese superoxide dismutase (SOD2) by means of acetylation and SIRT3-dependent deacetylation in response to oxidative stress. SOD2 is an important antioxidant enzyme and is found to be able to be acetylatedat lysine 68, leading to a significant decrease in its activity while mitochondrial deacetylase SIRT3 binds to, deacetylates, and activates SOD2. This study provided the evidence that increase of ROS levels stimulated SIRT3 transcription, leading to SOD2 deacetylation and activation to reduce ROS levels [107].

In the heart, the redox state in myocardial cells affects the cardiovascular function [108]. Recently, Ago et al. revealed that thioredoxin 1 (Trx1) could attenuate cardiac hypertrophy via inhibiting ROS-induced nucleocytoplasmic shuttling of class II HDACs [109]. This finding indicates that acetylation of histone contributes to aberrant redox state mediated cardiac hypertrophy. Furthermore, it has been reported that nicotinamide adenine dinucleotide phosphate oxidase 4 (Nox4) is involved in the regulation of HDAC4 nuclear exit, thereby mediating cardiac hypertrophy in response to ROS-generating hypertrophic stimuli, phenylephrine [110].

In conclusion, the epigenetic changes could represent an important pathway by which redox/oxidative metabolism affects the CVDs; however, there remains no much specifical information regarding the cardiac effect of these HATs and/or HDACs on the CVDs, which urgently needs to be further investigated for better understanding of the mechanisms for the development and progression of these diseases.

## 4. Clinical Translation and Application 

Research on histone acetylation and deacetylation covers broad clinical application, such as cancer [111], rheumatoid arthritis [112], Alzheimer's disease [113], stroke [114], and heart disease. There are many epigenetic treatment options currently used in the clinic that have been approved by the FDA, particularly for cancer treatment which have been shown to have a positive curative effect. However, in the treatment of cardiovascular disease, there are limited epigenetic treatment options available. A large number of studies indicate that epigenetic regulation of acetylation status shows high potential for clinical application in the diagnosis and treatment of cardiovascular diseases. The studies described in this review provide a basis for the evaluation of histone acetylation and deacetylation in patients with cardiovascular related diseases. However, more research is necessary to prove clinical benefit of the use of epigenetic drugs in human disease. By developing a new generation of treatment for cardiovascular diseases using epigenetic drugs, there is great potential to improve the quality of life of millions of CVD patients worldwide. This question is worth further investigation.

## 5. Summary

Reversible protein acetylation provides a central mechanism for controlling gene expression and cellular signaling events in cardiovascular and related diseases. Recent research has focused on histone modifications to provide a reliable theoretical basis for clinical treatment. A comprehensive understanding of acetylation mechanisms may lead to the development of novel and targeted treatment options for cardiovascular and related diseases.
